# Two case reports: EML4-ALK rearrangement large cell neuroendocrine carcinoma and literature review

**DOI:** 10.3389/fonc.2023.1227980

**Published:** 2023-11-01

**Authors:** Qin Chen, Jingjing Zhang, Xuan Wang, Wenkang Zong, Leina Sun, Jianwen Qin, Yan Yin

**Affiliations:** ^1^ Department of Respiratory and Critical Medicine, Tianjin Chest Hospital, Tianjin, China; ^2^ Department of Neurosurgery, Tianjin, China; ^3^ Department of Pathology, Tianjin Chest Hospital, Tianjin, China; ^4^ Department of Pathology, Tianjin Medical University Cancer Institute and Hospital, Tianjin, China

**Keywords:** large cell neuroendocrine carcinoma, EML4-ALK rearrangement, ALK-TKI inhibitor, alectinib, immunohistochemistry

## Abstract

Anaplastic lymphoma kinase gene (ALK) rearrangement is present in only approximately 5% of non-small cell lung cancers (NSCLCs) and is scarce in LCNEC patients. The conventional first-line treatment options are chemotherapy combined with immunotherapy or chemotherapy followed by palliative radiotherapy. In this report, we present two cases of metastatic LCNEC with EML4-ALK fusion that were treated with ALK-TKI inhibitors and demonstrated a rapid therapeutic response. Both patients were nonsmoking women who declined cytotoxic chemotherapy, underwent Next-Generation Sequencing (NGS), and confirmed EML4-ALK fusion. They were treated with alectinib as first-line therapy, and the tumors showed significant shrinkage after two months, achieving a PR (defined as a more than 30% decrease in the sum of maximal dimensions). The PFS was 22 months and 32 months, respectively, until the last follow-up. A systematic review of all previously reported cases of LCNEC with ALK mutations identified only 21 cases. These cases were characterized by being female (71.4%), nonsmoking (85.7%), diagnosed at a relatively young age (median age 51.1), and stage IV (89.5%), with an overall response rate (ORR) of 90.5%. PFS and OS were significantly longer than those treated with conventional chemotherapy/immunotherapy. Based on the clinical characteristics and the effective therapeutic outcomes with ALK inhibitors in LCNEC patients with ALK fusion, we recommend routine ALK IHC (economical, affordable, and convenient, but with higher false positives) as a screening method in advanced LCNEC patients, particularly nonsmoking females or those who are not candidates for or unwilling to undergo cytotoxic chemotherapy. Further molecular profiling is necessary to confirm these potential beneficiaries. We suggest TKI inhibitors as the first-line treatment for metastatic LCNEC with ALK fusion. Additional studies on larger cohorts are required to assess the prevalence of ALK gene fusions and their sensitivity to various ALK inhibitors.

## Highlights

1. EML4-ALK+ Neuroendocrine lung cancer treated with ALK-TKI inhibitors showed a rapid response;
2. Large cell Neuroendocrine cancer patients with ALK fusion are mostly nonsmoking, young, and female;
3. Routine ALK IHC is recommended in advanced large-cell neuroendocrine cancer patients.

## 1 Introduction

Pulmonary large cell neuroendocrine carcinoma (LCNEC) is a rare subtype of the primary tumor in the lung, with poor prognosis and rapid progression, and is prone to develop distant metastases, accounting for 2.1-3.5% of lung cancer (1). However, the incidence appears to be higher than the data because of difficulties in diagnosis based on cytological specimens (2). Most of the patients are elderly males who smoke (2–4) and are found to be in advanced stages with fewer opportunities for surgery. The conventional first-line treatment options in metastatic LCNEC patients are chemotherapy combined with immunotherapy or palliative radiotherapy. Because of the extremely low likelihood of finding a targetable genetic alteration, patients with LCNECs are not recommended for genetic testing in line with current guidelines unless they present with unusual clinical features that could hide an oncogene addiction, such as young age and no-smoking status. Due to its low mutation potential, there have been limited advances in targeted therapy research on LCNECs (5–7).

LCNECs are widely known to share unique molecular features, including abnormal gene status of TP53 and RB1 as well as high expression of tumor suppressor genes such as STK11, PTEN, KEAP1, and genes of the PI3K/AKT/mTOR pathway or RAS-pathway (KRAS/NRAS/HRAS) (5, 6, 8). LCNECs are reported to be strictly related to a heavy smoking habit (6, 9, 10). Rekhtman (11) proposed that LCNECs are divided into three subtypes with the presence or absence of RB1+TP53 alteration: SCLC-like and NSCLC-like subsets, respectively, and low total mutation burden defining carcinoid-like subsets.Anaplastic lymphoma kinase gene (ALK) rearrangement is present in only approximately 5% of NSCLCs and is even rare in NECLC patients (12). Nevertheless, this diagnosis gives patients an additional opportunity for targeted therapy. We describe 2 cases of metastatic LCNEC in female patients with EML4-ALK rearrangement who were administered ALK-targeted treatment and showed a partial response over 1-2 years.

## 2 Case presentation

### 2.1 Case 1

**Figure 1:**
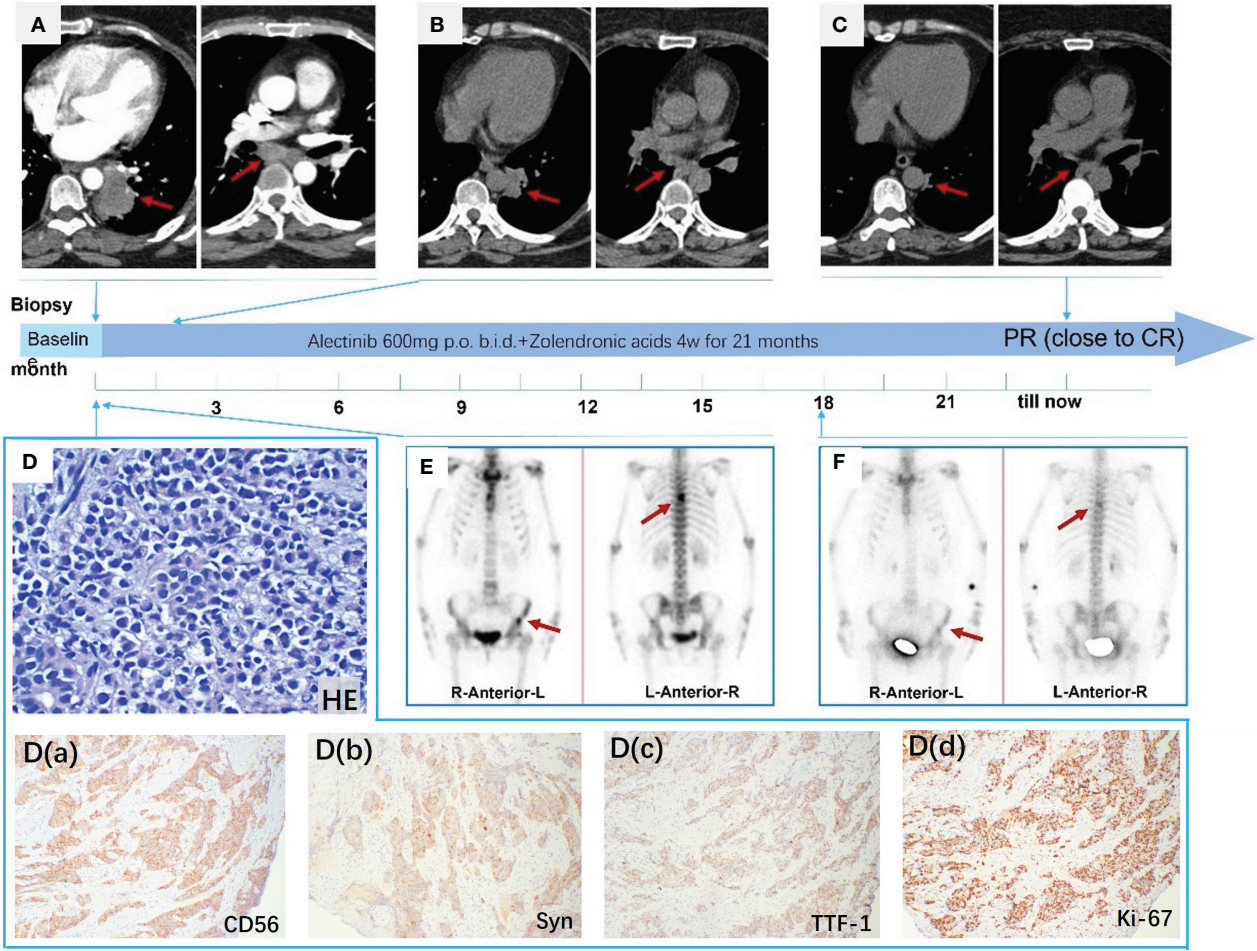
**(A)** CT enhancement scan shows a primary tumor in the left hilum with mediastinal and left hilar lymph node metastasis; **(B)** primary lung lesions were reduced significantly after two months; **(C)** CT scan demonstrating a dramatic shrinkage of the primary tumor after taking alectinib for 21 months; **(D)** HE staining at 100× magnification; **(D)** (a-d) Immunohistochemical stain revealed that CD56 (+), Syn (+), TTF-1 (+), and Ki-67 (+) (80%), ×100; **(E)** ECT showed the 6th thoracic vertebra and left localized iliac metastases; **(F)** distinctly decreased uptake of 99mTc-MDP in bone metastases after taking alectinib for 18 months.

A 53-year-old, nonsmoking married female with no family history of cancer death presented to Tianjin Chest Hospital with a 7-month history of “interrupted cough and wheezing.” Laboratory tests revealed abnormally elevated levels of neuron-specific enolase (NSE) (23.79 ng/ml; normal range 5.00–15 ng/ml). Contrast-enhanced chest CT showed a tumor in the left hilum (**Figure 1A**) with mediastinal and left hilar lymph node metastasis. A CT-guided lung biopsy was performed, along with baseline whole-body tumor burden and bone scanning (**Figure 1E**). Based on the abnormal radiological findings of lung tumor and bone metastasis, a diagnosis of metastatic pulmonary LCNEC was made according to hematoxylin and eosin (HE) staining (**Figure 1D**), positive immunoreactivity to CD56, Syn, and TTF-1, and a Ki-67 index of 80% [**Figure 1D** (a-d)]. The patient was considered to have advanced NECLC with multiple bone metastases, cT3N2M1c, and Union for International Cancer Control (UICC) Stage IVB, according to TNM Staging System 8th Edition (2017). Surgery was not appropriate. The patient declined chemotherapy, so we performed NGS (168 cancer gene panel; Burning Rock Dx, Guangzhou, China) using tissue biopsy to explore the mechanism and potential treatments. NGS identified an EML4-ALK rearrangement (E6:A20), with a PD-L1(22C3) TPS of 0%. The ALK inhibitor alectinib was prescribed at 600 mg twice daily after obtaining patient consent, and zoledronic acid was administered to suppress the progression of bone metastasis. The patient tolerated the treatment well; no grade 2–4 toxicities were noted. After two months of treatment with alectinib, the primary lung lesions responded significantly to treatment (**Figure 1B**), and the subsequent CT scan showed a durable response in the primary tumor. Additionally, there was a dramatic reduction in 99mTc-MDP uptake in bone lesions 18 months later (**Figure 1F**). The irritatingly interrupted cough and wheezing symptoms were also significantly reduced as the lung mass shrank significantly. Consequently, the patient’s quality of life improved after the treatment. The patient receives alectinib without evidence of disease progression with PFS for 21 months (**Figure 1C**).

### 2.2 Case 2

**Figure 2:**
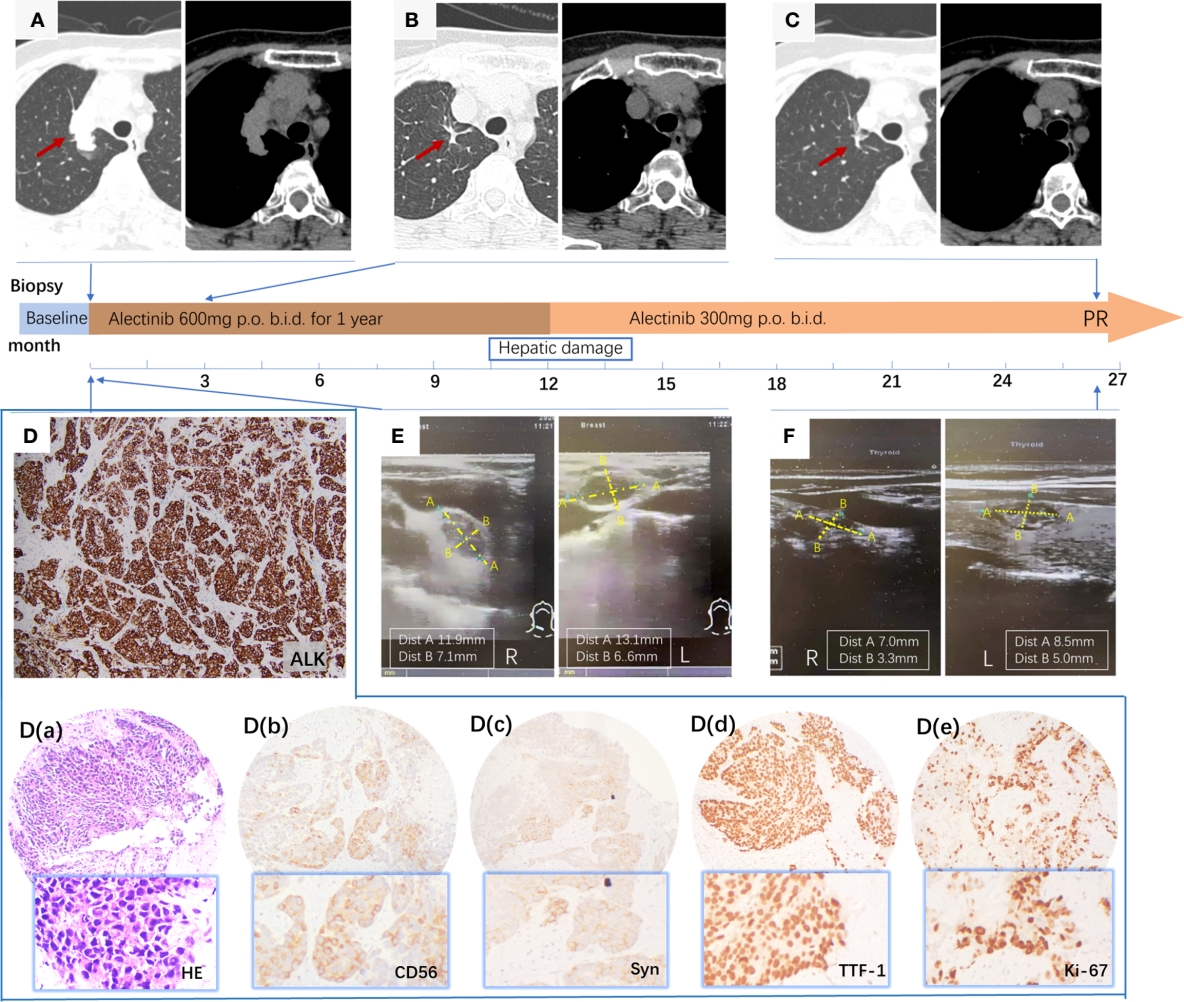
**(A)** Primary tumor in the right upper lung field; **(B)** Primary lung lesions distinctly shrink in 2 months; **(C)** Primary tumor showed a further decrease and almost disappeared after taking alectinib for 32 months; **(D)** positive ALK protein expression by IHC staining using the anti-ALK antibody (clone D5F3) characterized by intense, diffuse cytoplasmic staining; **(D)** (a-e) a, HE staining at 100× magnification; (b-e) Immunohistochemical stain revealed that CD56 (+), Syn (+), TTF-1 (+), and Ki-67 (+) (90%), ×100; **(E)** ultrasonography (USG) of bilateral superficial cervical lymph nodes metastasis; **(F)** USG of bilateral superficial cervical lymph nodes shrank obviously after 20 months of therapy.

A 62-year-old, never-smoking female with an unremarkable medical history presented to Tianjin Cancer Hospital with a palpable mass in the right supraclavicular fossae. Chest CT revealed a 4 cm irregularly shaped solitary tumor in the right upper lung field (**Figure 2A**). Ultrasonography (USG) of the superficial cervical lymph nodes (LNs) detected supraclavicular lymph node metastasis (**Figure 2E**) but no thyroid gland lesion. An ultrasound-guided node biopsy was performed on 14/9/2020, and the pathological examination showed metastatic large-cell neuroendocrine carcinoma characterized by IHC positivity for CD56(+), thyroid transcription factor (TTF-1), synaptophysin (Syn), and diffuse expression of ki-67. The Ki-67 index was 90% [**Figure 2D** (a-e)]. HE staining under the microscope revealed distinct neuroendocrine differentiation, characterized by non-small cell morphology, abundant cytoplasm, and well-defined nucleoli [**Figure 2D** (a)]. Given the patient’s never-smoker status, a molecular work-up for potentially actionable mutations was conducted using Ventana ALK IHC (D5F3), which unexpectedly revealed positive staining for ALK (**Figure 2D**), which was confirmed by NGS, revealing an EML4-ALK rearrangement (E20:A20). The patient was diagnosed with LCNEC of the lung, UICC stage IIIB, according to the TNM Staging System 8th Edition, 2017. The patient immediately started on second-generation ALK inhibitor alectinib at 600 mg b.i.d. in October 2020. The patient showed a rapid excellent tumor response to alectinib, with shrinkage of the pulmonary nodule in 2 months (**Figure 2B**). However, after one year of therapy, the patient experienced side effects of abnormal liver function. A halved dose reduction was performed, and liver function promptly recovered. Despite the reduced alectinib dose, the patient continued demonstrating sustained clinical, radiographic, and biochemical responses while on alectinib for 32 months at writing, without any other drug-related toxicities (**Figures 2C, F**).

## 3 Discussion

**Figure 3:**
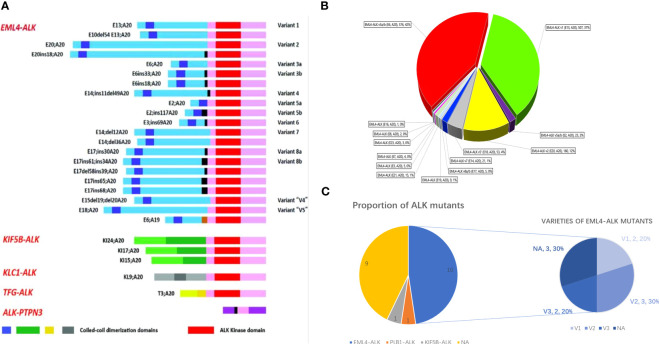
**(A)** Schematic of various ALK fusion variants in NSCLC, adapted from Ou et al. (29). **(B)** V proportion of 1387 EML4-ALK variants in EML4-ALK+ NSCLC, adapted from Zhang et al. (30). **(C)** Pie chart of ALK variants detected in LCNEC. The left graph shows the prevalence of fusion partners reported in our 19-case review, and the right diagram depicts the distribution of different EML4-ALK variants within the limited 9 cases.

Lung neuroendocrine tumors account for approximately 20% of all lung cancers. They can be categorized into typical carcinoid (TC 1.8%), atypical carcinoid (AC 0.2%), large cell neuroendocrine carcinoma (LCNEC, 3%), and small cell lung cancer (SCLC, 15%) (13, 14). LCNEC, representing a small proportion of neuroendocrine lung cancers, is typically associated with a high rate of metastasis and poor survival, but the survival ranges are notably broad (11) (**Figure 3A**). Although the ALK mutation is relatively common in NSCLC, it is an extremely rare subtype in pulmonary neuroendocrine tumors. We have reviewed the data from one of our tumor centers (Tianjin Chest Hospital, where case 1 came from), which treated approximately 22,400 lung cancer patients from January 2018 to December 2022. Of these, 515 cases (2.3%) were diagnosed with LCNEC, 31 of which were composite LCNEC (composed of LCNEC and other types of cancer like squamous cell carcinoma or adenocarcinoma). Unfortunately, we have only identified one case of LCNEC with ALK mutation. We believe that the true incidence of LCNEC is higher than the current literature reported data, as cytological specimens of lung LCNEC are relatively difficult to diagnose. The most significant molecular profiling study on neuroendocrine lung cancer involved a retrospective cohort of 439 lung neuroendocrine neoplasms (L-NENs) patients (12), in which ALK rearrangements were identified in 4 cases (3 out of 61 LCNEC and 1 out of 69 AC cases). In two additional studies that included 63 LCNEC or 219 L-NENs cases (78 of which were LCNEC), no ALK rearrangements were detected, including fusion, mutation, and copy number amplification (6, 15). However, in an additional genomic profiling study of 108 L-NENs cases, among which 52 were LCNEC, one ALK rearrangement was detected in an LCNEC patient (8).

Our current review provides the most comprehensive overview of ALK mutations in LCNEC to date. We have leveraged two of the most reputable medical databases, PubMed and Web of Science, to identify all reported LCNEC cases with ALK mutations meticulously. As a result of our comprehensive literature review, 19 patients of LCNEC with ALK mutations have been documented to date (summarized in **Table 1**). The majority of these mutations were screened utilizing immunohistochemistry (IHC), with confirmation by fluorescent *in situ* hybridization (FISH) or molecular techniques, such as RNA-based PCR or next-generation sequencing (NGS).

**Table 1:** Cases of patients with ALK-fusion LCNEC from the literature review.

Pathology	Ref.	Age(yr)	Sex	Smoke	Sampling	Size	Stage	Metastasis	Testing Method	gene mutation	treatment	PFS(mon)
1 LCNEC	2002 (12)	37	M	NO	Brain metastases resection	1 cm	cT1cN3cM1c	Multiple (more than 50) brain metastases	IHC (3+) NGS	EML4-ALK (V3a/b) CDKN2A/B	Alectinib 10 m Lorlatinib 12 m	22
2 LCNEC	2022 (12)	32	F	NO	ovariectomy	NA	cT2cN1pM1c	Brain/ovary	NGS	EML4-ALK (V1) CDKN2A/B loss	EC NE Alectinib	5*
3 LCNEC	2022 (12)	68	F	NO	NA	NA	cT3cN3cM1a	pleural/bone/liver/brain	IHC (3+)-NGS	EML4-ALK (V1) CDKN2A/B loss MTAP loss JAK2 V617F	EC*6-NE Alectinib	PD
4 LCNEC	2020 (16)	32	F	YES	Lung biopsy EBUS-TBNA(LN), breast tumors and liver	3 cm	pT1N2M1c	Bone/brain/breast/liver	IHC-FISH(58%)	ALK-rearrangement (all)	Alectinib	11
5 LCNEC	2022 (17)	38(pregnant)	F	NO	Brain metastases resection	5 cm	pT2N0M1c	Brain/adrenal gland	CtDNA NGS	ALK-EML4	Alectinib	10*
6 LCNEC	2021 (18)	37	M	NO	Liver biopsy	5.1 cm	pT3N3M1c	Lung/Brain/bones/liver	IHC(3+)-NGS ctDNA NGS	1st:EML4-ALK 2nd: ctDNA ALK V1180L; G1202R; TP53; G105V; MYCN E378fs	Alectinib 4 m RT*+brigatinib; PD lorlatinib PD CE-death	4
7 LCNEC-CM	2021 (19)	58	F	NO	EBUS-TBNA(LN)	NA	T2N2M0		IHC-FISH	ALK rearrange	CE-6 m stereotaxic radiation PD Alectinib 4 m	4 PD
8 LCNEC-CM	2021 (19)	74	F	NO	Transbronchial Biopsy	NA	T2N2M1	Bone	IHC-FISH	ALK rearrange	CE-NE Crizotinib 11 m Ceritinib 12 m Brigatinib 33 m	56*
9 LCNEC	2021 (19)	34	F	NO	Transbronchial Biopsy	NA	TxNxM1	Bone/brain	IHC-FISH	ALK rearrange	Alectinib(side effect) Crizotinib 4 m	4 death
10 LCNEC	2021 (20)	41	M	YES	Lung biopsy	4.2 cm	T2N2M1c	Brain	IHC-FISH(56%)	PLB1-ALK (P56:A19)	EC*2 PD Crizotinib 5 m Ceritinib 4.5 m PD	9.5
11 LCNEC	2021 (21)	72	M	NO	Brain metastases resection	3.8 cm	T2N2M1c	Liver/adrenal gland/brain	IHC-FISH (96%)	ALK rearrange	Alectinib	4
12 LCNEC	2019 (22)	51	M	NO	Lung biopsy	3.4 cm	T2NxM1c	Bone/brain	IHC-FISH	ALK rearrange	Crizotinib	11
13 LCNEC	2018 (7)	75	F	NO	Transbronchial Biopsy	2.1 cm	T1cN2M1c	Bones/livers	FISH	ALK rearrange	Alectinib	6*
14 LCNEC	2019 (23)	73	M	NA	Thyroidectomy	3.2 cm	T2N0M1c	Bone/thyroid	IHC-FISH rt-PCR	KIF5B-ALK	Crizotinib10 m (brain met) Alectinib 4 m+	14*
15 LCNEC	2018 (24)	58	F	NO	NA	NA	cT2N2M1c	Brain/liver	IHC-FISH NGS	EML4-ALK METexon14 R988C	Crizotinib	5*
16 LCNEC	2014 (25)	43	F	NO	Breast resection, Transbronchial Biopsy, skin biopsy	5 cm	T2NxM1c	Breast/skin/brains/bone/liver	IHC-FISH (All three obtain samples (+)	EML4-ALK(V2)	Crizotinib	PD
17 combined AC and LCNEC	2022 (26)	61	F	NO	Bronchoscopy+lobectomy +bone biopsy	2.5 cm	pT2aN1M1c	Bone (vertebra and iliac)	FISH	ALK rearrange (both)	EC NE Alectinib	6*
18 LCNEC	2022 (27)	47	F	YES	Bronchoscopic biopsy	NA	PTxN3M1c IV	Brain/adrenal gland/liver	NGS	EML4-ALK V2(E20:A20); 3^rd^: V1180L; 4^th^: L1196M+CDKN2A; 5^th^: p.L1196M/p.G1202R +p.L1196M/p.D1203N	1^st^: Crizotinib 3m (breast met) 2^nd^: Alectinib 10 m+ 3^rd^Ceritinib without success; 4^th^: brigatinib or carboplatin/pemetrexed; 5^th^:lorlatinib 8m+	37mon
19 LCNEC	2023 (28)	57	F	YES	Bronchoscopic biopsy	3 cm	cT2aN2M0 IIIa	NO	NGS+IHC+FISH	ALK rearrangement	1^st^: Alectinib 3m (brain met) 2^nd^: Brigatinib without success; 3^rd^: lorlatinib 21	24mon
20 LCNEC	Case1	53	F	NO	Lung biopsy	6 cm	cT3N2M1c	Bone (vertebra and iliac)	NGS	EML4-ALK (E6:A20)	Alectinib	21*
21 LCNEC	Case2	63	F	NO	LN Biopsy	4 cm	T2N3M0	NO	IHC+NGS	EML4-ALK (E20:A20)	Alectinib	32*

*No progress until the last follow-up; NE, not effective; NA, not available; RT, radiotherapy; CM, carcinoid morphology; AC, adenocarcinoma; FISH, fluorescence in situ hybridization; IHC, immunohistochemistry; NGS, next-generation sequencing; rt-PCR, real-time fluorescence polymerase chain reaction; PFS, progression-free survival.

We have summarized the clinical characteristics of ALK+LCNEC cases (**Table 2**) and evaluated the therapeutic efficacy of targeted treatment. Additionally, we have highlighted several issues related to the clinical management of these patients for further discussion.

**Table 2:** Clinicopathological characteristics of the retrospective cases (n=19) and patient index cases (n=2).

Characteristics	N=21
Age, median (range) (ys)	51 years (range 32-75)
<60	14 (66.7%)
>60	7 (33.3%)
Histological subtype, n (%)	
LCNEC	18 (85.7%)
LCNEC-CM	2 (9.5%)
LCNEC combined with AD	1 (4.8%)
Sex, n (%)	
Male	6 (28.6%)
Female	15 (71.4%)
Smoking status, n	
Never	16 (76.2%)
Former/current	4 (19.0%)
NA	1 (4.8%)
TNM Stage, n (%)	
I-III	3 (14.3%)
IV	18 (85.7%)
Metastasis	
Brain	13 (61.9%)
Bone	11 (52.4%)
Liver	8 (38.1%)
Adrenal gland	3 (14.3%)
Others (ovaries/breast/thyroid)	1 (4.8%)/2 (9.5%)/1 (4.8%)

CM, carcinoid morphology; AD, adenocarcinoma; NA, not available.

### 3.1 Clinical characteristics of patients with LCNECs and ALK rearrangements

A summary of the clinical characteristics of the 21 LCNEC patients in this series, including the two cases mentioned previously, is provided in **Table 2**. Out of the 21 patients, 15 (71.4%) were female. The average age at diagnosis was 51.0 years, ranging from 32 to 75 years. Among the 20 patients with known smoking status, 16 (76.2%) had neither a current nor a previous smoking history. Most cases (18 out of 21, 85.7%) were diagnosed at a metastatic stage, with 61.9% (13/21) of these cases associated with brain metastases.

The oncogenic or targetable genetic ALK alterations identified during our case review included ten cases of EML4-ALK fusion, one case of KIF5B-ALK fusion, and one case of PLB1-ALK fusion. The remaining nine cases with ALK mutations did not have specific details about the mutation type due to the limitations of the detection methods (**Figure 3C**). Only two patients were identified with ALK drug-resistant mutations (V1180L, G1202R, L1196M, and D1203N) following the initial ALK-targeted treatment (18, 27). The disease control rate (DCR) was assessed in all 21 patients, resulting in a rate of 90.5% (19 in 21 cases). Only one patient treated with alectinib experienced Grade 3 side effects and was subsequently switched to crizotinib (19). We further analyzed the median period of PFS for all available cases, reaching a result of 14.03 ± 0.37 months. The actual PFS is likely to be more optimistic than this due to the limitation of post-publication follow-up in some case reports.

Five of the 13 patients with brain metastases were initially treated with crizotinib, while the other eight received alectinib as their first-line therapy. Both treatment regimens were effective, except for three patients experiencing PD within three months (12, 25, 27). In total, 11 patients benefited from second-line ALK TKIs, such as ceritinib, brigatinib, and lorlatinib. One case successfully underwent third-line therapy with ALK inhibitors (19). Unfortunately, further details about the resistance mechanism in this case were not provided. In another case report, a patient received a prolonged treatment regimen up to the 5th line of ALK-TKI therapy. After eight months of progression-free survival (PFS) following the 5th line of lorlatinib treatment, multifocal progression occurred. The patient’s NGS results revealed the presence of ALK:p.L1196M/p.G1202R and p.L1196M/p.D1203N mutation, indicating resistance to all currently marketed ALK-TKI inhibitors.

The above cases emphasize the importance of obtaining histological specimens from biopsies, as molecular profiling, including IHC, FISH, and NGS, is highly dependent on these specimens to develop targeted therapy plans. This highlights the actual value of histology in lung cancer during the prevalent era of targeted therapy, which radically changes the therapeutic approach to lung cancer.

Our findings revealed that LCNEC patients with ALK mutations tend to be relatively young, non-smoking females. The most common mutation type was EML4-ALK fusion. Most of these patients were likely to have advanced-stage disease at diagnosis, characterized by multiple metastatic sites, particularly in the central nervous system (CNS). Nonetheless, ALK inhibitors proved effective and safe treatment, even when used in sequential prescriptions.

### 3.2 Testing algorithm for detecting ALK mutations in LCNECs

Various methods are currently available for detecting ALK mutations, each with merits and limitations. FISH, IHC, RT-PCR, and NGS are frequently employed on tissue samples. FISH remains the established benchmark for diagnosing ALK fusions in NSCLC. The FDA-approved Ventana ALK IHC (D5F3) also plays a role in therapeutic decision-making. However, both IHC and FISH rely heavily on the pathologist’s interpretation of the results, and they have a small likelihood of false positives due to signal instability and low sensitivity, which could result in a few patients missing treatment opportunities due to these false negatives. The advantages of NGS detection lie in its ability to sequence millions to billions of DNA fragments simultaneously, allowing for cost-effective detection of upwards of hundreds of tumor-related genes at a lower cost. This approach eliminates the need for preliminary knowledge of potential tumor mutation types, enabling result-oriented detection methods such as IHC and FISH (31). It can also reveal resistance mechanisms to targeted drugs (32, 33), particularly for patients with rapidly worsening conditions, thereby guiding clinical decisions at the earliest opportunity (34).

The evaluation of the techniques above primarily focuses on the methodological aspects; however, it is essential to integrate them with the patient’s actual clinical condition to establish an appropriate testing strategy. A significant case series study by Qiang Zheng screened the ALK status in all L-NENs and found that the frequency (2.7%) was slightly lower than that in adenocarcinoma (3%–5%) (35). Another study reviewing the most significant retrospective cohort of 436 lung NECs reported a 9.7% positive staining rate for ALK by IHC (Clone 5A4; Leica, Wetzlar, Germany), with only 4 cases confirmed by FISH or NGS (12). Kondoh evaluated ALK expression (Clone 5A4; Abcam, Cambridge, UK; Dako Flex+ method, Copenhagen, Denmark) in 142 SCLCs and found an 11.3% positive rate, all of which were negative by FISH or RT-PCR (36). The expression pattern of ALK in SCLC differs from that in adenocarcinoma. In contrast to strong and uniform expression in adenocarcinoma, the intensity and proportion of ALK expression vary between individual tumors, and all ALK IHC-positive tumors expressed ALK in some tumor cells. This signifies a distinction between NSCLC and SCLC in the mechanism of ALK expression, which might be associated with neuroepithelial features of NENs. The suggestion is made that the expression of ALK is unlikely to be used as a molecular target, given its physiological expression in neurocytes. Takeuchi et al. suggested that IHC-detected ALK fusion could lead to higher false positives in some carcinomas with neuroendocrine differentiation, particularly in LCNEC, due to the intrinsic expression of standard ALK transcript. ALK IHC in treatment-naive LCNECs should not be employed as a predictive biomarker for ALK inhibitor therapy, resulting in a high prevalence of false-positive cases and low specificity. This contrasts with the view that ALK positivity by Ventana ALK IHC (D5F3) is typically considered diagnostic and prompts targeted treatment with ALK inhibitors in NSCLC (37, 38).

**Figure 4:**
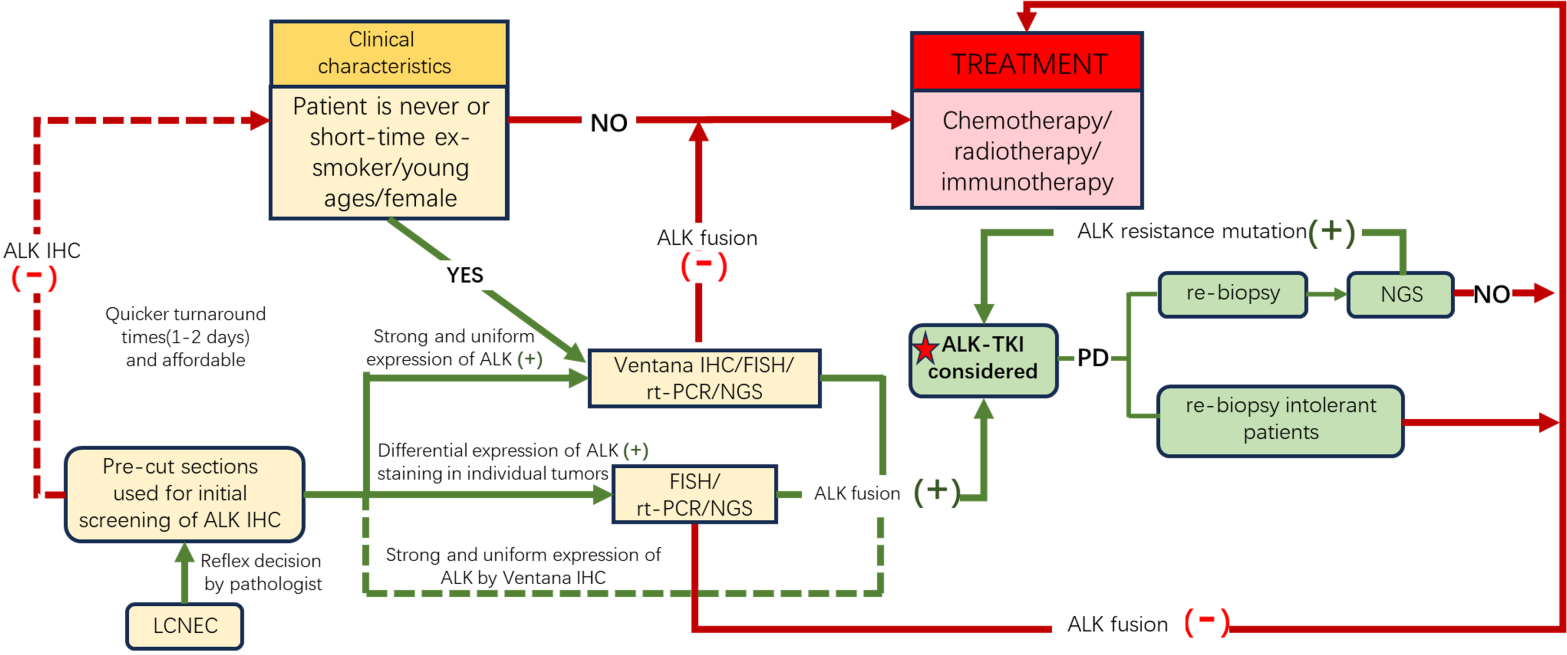
A possible molecular testing algorithm for LCNEC patients. Given the physiological expression of ALK in neurocytes, we recommend performing ALK IHC to screen out potential candidates (yellow boxes), and for those with IHC ALK+ or with clinical features of nonsmoking history, young age, or female sex despite negative ALK, further molecular tests should be conducted to confirm ALK fusion to direct precision therapy. Furthermore, re-biopsy should be performed after first-line or second-line ALK-TKI resistance. The yellow boxes reveal the potential group benefit from ALK-TKIs. The red box and lines show patients who received traditional treatment. Green boxes and lines indicate the population that benefited from ALK-TKIs. ALK, anaplastic lymphoma kinase; TKI, tyrosine kinase inhibitor; FISH, fluorescence *in situ* hybridization; IHC, immunohistochemistry; rt-PCR, real-time polymerase chain reaction; NGS, next-generation sequencing.

Therefore, we propose a potential molecular testing algorithm for effectively screening LCNEC patients with ALK rearrangement (**Figure 4**). Despite the advantages of NGS in throughput and visualization, IHC remains the quintessential screening tool for ALK-rearranged carcinoma. We recommend employing IHC (Ventana ALK IHC (D5F3) is suggested) to identify candidates for ALK inhibitor treatments, followed by a confirmatory process, such as NGS or ALK FISH, in ALK IHC-positive patients. However, in cases of LCNEC patients with clinical characteristics of younger age or nonsmoking females, further NGS/FISH/rt-PCR should be performed regardless of the IHC findings.

Obtaining more compelling data to support and optimize this algorithm could be challenging due to the rarity of this finding. However, it is essential for improving diagnostic accuracy and guiding appropriate treatment strategies.

### 3.3 ALK rearrangements and treatment strategies in LCNECs

ALK was initially identified as a fusion gene in anaplastic large-cell lymphoma (ALCL) (39). With the advent of deep sequencing methods, the number of distinct fusion partners identified in ALK+ NSCLC had reached 90 by the end of January 2020, according to the latest data from JTO (40). In addition to the EML4-ALK fusion, which accounts for approximately 85% of the fusion variants, other ALK fusions, such as PTPN3, TFG, KIF58, KLC1, STRN, ERCC8, DCTN1, and STRN, etc., have also been reported (**Figure 3A**). Most of these novel ALK fusion variants have been found to respond to ALK TKIs (40–42). The dominant EML4-ALK can be further subdivided into “short” (v3a/b and v5a/b, without the TAPE domain) and “long” variants (v1, v2, and v4, with the parts of the TAPE domain), leading to varying degrees of protein stability and ultimately different responses to ALK inhibitors (30) (**Figure 3B**). Horn and Lovy conducted a comprehensive analysis of the cellular inhibitory activity of all six ALK inhibitors. They found that specific resistance mutations, such as ALK G1202R, G1202del, and ALK L1198F, confer higher resistance levels (IC50) to brigatinib and lorlatinib in the V3 background. Therefore, the fusion variant background should be considered when interpreting ALK resistance mutations (43).

In our literature review, only 10 cases provided detailed information on EML4-ALK variant subtypes. Omachi reported an LCNEC case with a poor prognosis and limited therapeutic responses to ALK inhibitors, specifically harboring the V2 variant (25). In contrast, patients with the V3 variant, reported by Akhoundova or the index case 1 patient, benefited from alectinib, with 22 or 14 months of PFS, respectively (up to the time of publication). These cases seem to contradict the general opinion in NSCLC that shorter variants exhibit a decreased response to ALK inhibitors. Therefore, gathering more patient samples to compare variant expression, subsequent response, and long-term responses to different TKI regimens is crucial. The characteristics of ALK mutations in LCNEC differ from those in NSCLC, as confirmed in our summary of 13 ALK+ LCNEC patients with intracranial metastasis, demonstrating weaker central nervous system efficacy.

Additionally, we investigated further research to identify exciting alternative treatment options that could extend PFS and OS in NSCLC and apply these findings to our LCNEC patients. Above all, it is essential to recommend re-biopsy or liquid gene testing for gene sequencing when patients develop PD to guide our subsequent treatment regimen (15, 44). This allows us to switch to newer generation ALK TKIs (45–49) or explore other strategies to overcome drug resistance in NSCLC (50–57).

Although most resistance mechanism studies and clinical data mentioned above are derived from NSCLC, our limited dataset of 21 LCNEC patients treated with ALK-targeted therapy suggests that they generally follow the same ALK+ pattern as NSCLC. However, due to the limited data on ALK+LCNEC patients, we exercise caution when interpreting these results. We eagerly await additional basic research and large-sample meta-analyses to further elucidate the mechanisms and characteristics of ALK TKI inhibitor resistance in LCNEC patients.

## 4 Conclusion

LCNEC patients with ALK mutations are a more aggressive subtype based on our literature review, but most of them could benefit from ALK inhibitors to prolong survival duration. Given the genomic heterogeneity of LCNEC, we recommend implementing routine ALK IHC testing in advanced LCNEC patients, particularly younger and nonsmoking females, to identify potential candidates. Additionally, integrated with FISH or NGS, comprehensive molecular profiling is necessary to confirm the benefits of ALK IHC-positive patients, all predicated on obtaining patient histology. These findings create avenues for personalized treatment in LCNEC, which may lead to a more sustained response and increased long-term survival.

## Data availability statement

The original contributions presented in the study are included in the article/supplementary material. Further inquiries can be directed to the corresponding author.

## Ethics statement

The studies involving humans were approved by Ethics Committee of Tianjin Chest Hospital:IRB number: 2023LW-002, the date of IRB approval: Feb 16th, 2023. The studies were conducted in accordance with the local legislation and institutional requirements. Written informed consent for participation was not required from the participants or the participants’ legal guardians/next of kin in accordance with the national legislation and institutional requirements. Written informed consent was obtained from the individual(s) for the publication of any potentially identifiable images or data included in this article.

## Author contributions

QC: Original draft preparation, Investigation, Funding acquisition. JZ: Editing, investigation. XW: Validation and Investigation. WZ: Resources. LS: Conceptualization, Resources. YY: Conceptualization. JQ: Supervision, Review & Editing. All authors contributed to the article and approved the submitted version.
